# Caspases play in traffic

**DOI:** 10.1038/cddis.2017.55

**Published:** 2017-03-02

**Authors:** Catherine M Duclos, Audrey Champagne, Julie C Carrier, Caroline Saucier, Christine L Lavoie, Jean-Bernard Denault

**Affiliations:** 1 Department of Pharmacology-Physiology and Institut de Pharmacologie de Sherbrooke, Université de Sherbrooke, Faculty of Medicine and Health Sciences, 3001, 12th Avenue North, Sherbrooke, QC, Canada J1H 5N4; 2 Department of Anatomy and Cell Biology, Université de Sherbrooke, Faculty of Medicine and Health Sciences, 3001, 12th Avenue North, Sherbrooke, QC, Canada J1H 5N4

The unfolding of apoptosis involves the orchestrated proteolysis of hundreds of proteins,^1^ and to achieve efficacy the cell has evolved a three-step activation cascade. First, initiator caspases (caspases 8, 9, 10) are activated either by external death ligands triggering the formation of the death-inducing signaling complex (DISC; caspases 8 and 10) or by the release of cytochrome *c* from mitochondria provoking the assembly of the apoptosome (caspase-9).^2^ Once activated, the initiator caspases directly cleave executioner caspases 3 and 7, leading to their activation. The third step is the caspase-3-mediated activation of the executioner caspase-6, a peptidase with a substrate preference similar to that of initiator caspases. In this manner, the cell amplifies the proteolytic activity of caspases to swiftly cause cell demise.^2^

As caspases become active, proteolysis of various death substrates happens with the general goal of stopping crucial cellular processes, but for a limited number of proteins a gain in function occurs, often via the loss of a regulatory domain or the separation of domains carrying different functions. These substrates are difficult to identify as they necessarily require the study of individual fragments generated by caspases and because their proteolysis does not occur in isolation. Earlier studies have established that most caspases recognize a five-amino-acid motif in their substrates and cleave following an aspartate residue. However, recent reports have muddied the water in respect to these simple rules for substrate recognition and cleavage,^3, 4^ by showing that caspases can also cut after a glutamate residue^5^ and employ exosites to improve catalysis.^6^ It is therefore not trivial to assign a role to a specific cleavage event and a substrate to a specific caspase.

In a recent publication,^7^ we identified two novel caspase substrates, sorting nexin (SNX) 1 and 2, involved in endosomal sorting (Figure 1). The two proteins are cleaved by initiator caspases *in vitro* and *in cellulo* during apoptosis. We further showed that SNX1 contains multiple cleavage sites, including following glutamate residues. Interestingly, cellular repression of caspase-6, which also cleaves SNX2, did not fully abolish SNX2 proteolysis, suggesting that proteases upstream of this caspase may also participate in the cleavage of SNX2 in cells. Furthermore, only initiator caspases were able to cleave SNX1, reinforcing the idea that initiators contribute to the cleavage of SNX proteins in cell death. This possibility is of particular interest as few substrates have been assigned to initiator caspases.

SNX1 and SNX2 are two early endosome-localized proteins belonging to the SNX family of proteins, whose members are critical for protein trafficking steps within the endocytic pathway. Both SNX1 and SNX2 regulate the endosome-to-TGN (*trans*-Golgi network) retrograde transport of lysosomal receptors as part of the retromer complex (Vps26, Vps29, Vps35) and the endosomal sorting of various plasma membrane receptors. Our work demonstrates that SNX2 cleavage leads to dissociation from Vps35 and the delocalization of Vps26, suggesting the uncoupling of the endosomal cargo recognition machinery from the transport machinery itself. Consequently, the cleavage of SNX2 and by extension that of SNX1 affect the trafficking of several proteins.

One aspect that may be affected by SNX cleavage is apoptosis via death receptors that requires their trafficking in endosomes, where the associated initiator caspase-8 activates the bulk of the executioner caspase-3 (reviewed in Guicciardi *et al.*^8^). Moreover, studies have demonstrated that caspase-3 immobilization via palmitoylation at the plasma membrane, where caspase-8 is initially recruited at the DISC, enhances its activation. This suggests that co-localization of a caspase with specific substrates can result in more efficacious proteolysis. Although we have not yet assessed the role of SNX cleavage in modulating caspase-8-mediated apoptosis, this avenue is appealing in light of our findings because SNX1 and SNX2 promote endosomal trafficking. Thus, we speculate that SNX1 and SNX2 cleavage by caspase-8, which we demonstrated are best performed by this caspase *in vitro*, is a means to stall internalized DISC in vesicles and further promote the activation of caspases.

A second way SNX1 and SNX2 cleavage may affect the cell is by modulating the endosomal sorting of many signaling receptors, including receptor tyrosine kinases (RTKs). Significantly, we showed that cellular depletion of SNX2 increased hepatocyte growth factor (HGF) RTK (MET) phosphorylation and its capacity to support Erk1/2 activation, demonstrating the importance of SNXs' sorting function in the regulation of receptor signaling. We suggest that this becomes important in resistance to cancer treatment.^9^ Indeed, results from pre-clinical studies and clinical trials using death receptor agonists, such as the tumor necrosis factor-related apoptosis-inducing ligand (TRAIL) and antibodies, have been plagued by the emergence of resistance to such treatments.^10, 11^ Although several mechanisms may lead to TRAIL resistance, two mechanisms involve increased expression of anti-apoptotic or the downregulation of pro-apoptotic Bcl-2 family members,^11^ which regulate cytochrome *c* release from the mitochondrion. Another means to escape apoptosis is by boosting the X-linked inhibitor of apoptosis protein (XIAP) level, which in normal cells defines the threshold for apoptosis by inhibiting caspases 3, 7 and 9 directly.^12^ In the caspase activation cascade, these mechanisms of resistance lie between the activation of initiator caspases 8 and 10 and that of downstream effector caspases.

Based on these premises, our prediction is that death ligand-resistant cells should activate caspase-8, but not the executioners, and survive. This activation should also result in the cleavage of a limited set of caspase substrates. As a consequence, the cleavage of SNX2 (and SNX1) may be analogous to its depletion and results in significantly enhanced MET signaling, as we have shown. Furthermore, although the cell usually cleaves MET during apoptosis to inactivate it, this is a task likely performed by caspase-3, which is neither activated nor inhibited in death agonist-resistant cells. Therefore, we postulate that the activation of initiator caspases may enhance the metastatic potential of apoptosis-resistant cancer cells by the selective cleavage of proteins involved in RTK endocytic trafficking. In support of this idea, we showed that low SNX2 mRNA level in primary colorectal tumors is indicative of poor overall patient survival. These findings open new lines of inquiries into the role caspases play in RTK trafficking. Contrary to proteins directly involved in receptor signaling, these pathways are not typically plagued with mutations in cancer, and may, therefore, represent promising alternative targets for therapies.

## Figures and Tables

**Figure 1:**
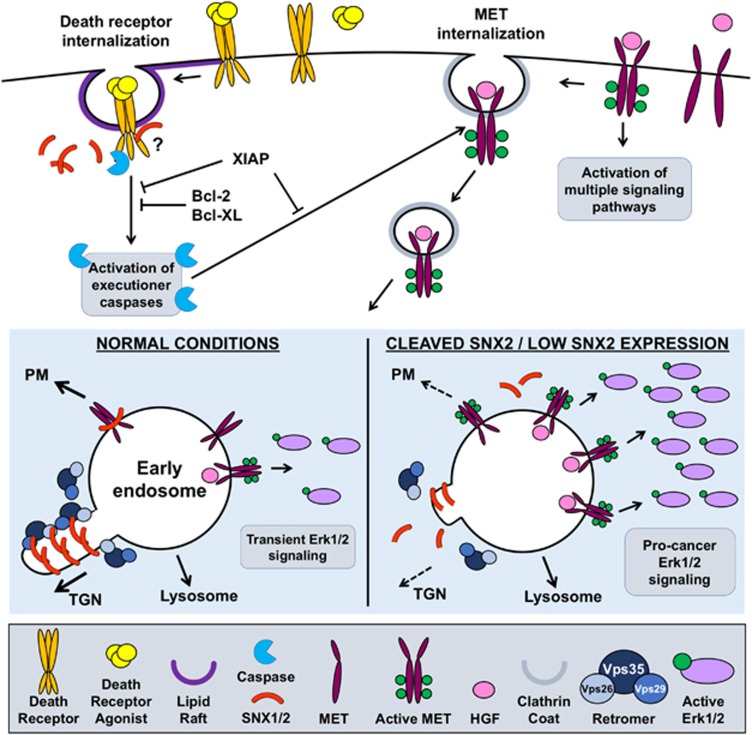
Schematic of caspase-mediated SNX1 and SNX2 proteolysis and its effect on MET signaling. The stimulation of death receptors causes their internalization via lipid rafts. Once internalized, sufficient caspase-8 activation occurs, allowing the activation of executioner caspases. The cleavage of SNX1 and SNX2 may promote caspase activation. HGF binding induces autophosphorylation of its receptor, promoting the activation of multiple signaling pathways. Internalized MET traffics to early endosomes (at least in Hela cells), where Erk1/2 activation occurs. In normal conditions, SNX1 and SNX2 associate with the retromer to mediate retrograde transport of cargo to the TGN and also participate in receptor recycling to the plasma membrane. During apoptosis, both SNX1 and SNX2 are cleaved, abrogating the interaction with the retromer, whereas low SNX2 expression exacerbates MET and Erk1/2 signaling with potentially detrimental effects

## References

[bib1] Crawford ED et al Mol Cell Proteomics 2013; 12: 813–824. 23264352 10.1074/mcp.O112.024372 PMC3591672

[bib2] Boucher D et al Encyclopedia of Signaling Molecules. Springer: New York, NY, USA, 2012, pp 242–256.

[bib3] Thornberry NA et al J Biol Chem 1997; 272: 17907–17911. 9218414 10.1074/jbc.272.29.17907

[bib4] Stennicke HR et al Biochem J 2000; 350: 563–568. 10947972 PMC1221285

[bib5] Seaman JE et al Cell Death Differ 2016; 23: 1717–1726. 27367566 10.1038/cdd.2016.62 PMC5041198

[bib6] Boucher D et al Proc Natl Acad Sci USA 2012; 109: 5669–5674. 22451931 10.1073/pnas.1200934109 PMC3326497

[bib7] Duclos CM et al Cell Death Discov 2017; 3: 16100. 28179995 10.1038/cddiscovery.2016.100 PMC5253419

[bib8] Guicciardi ME et al FASEB J 2009; 23: 1625–1637. 19141537 10.1096/fj.08-111005 PMC2698650

[bib9] Hanahan D et al Cell 2011; 144: 646–674. 21376230 10.1016/j.cell.2011.02.013

[bib10] Stuckey DW et al Trends Mol Med 2013; 19: 685–694. 24076237 10.1016/j.molmed.2013.08.007 PMC3880796

[bib11] Dimberg et al Oncogene 2013; 32: 1341–1350.22580613

[bib12] Jost et al Nature 2009; 460: 1035–1039.19626005

